# Host Specificity in Canopy Nesting Forms of *Ochrogaster lunifer*: The Larger Children Do Not Care

**DOI:** 10.3390/insects14050420

**Published:** 2023-04-27

**Authors:** Julianne Farrell, Myron P. Zalucki, Andrea Battisti

**Affiliations:** 1 School of Biological Sciences, University of Queensland, St. Lucia Campus, Brisbane, QLD 4072, Australia; juliannefarrell17@gmail.com (J.F.); m.zalucki@uq.edu.au (M.P.Z.); 2 Department DAFNAE, University of Padova, Agripolis Campus, 35020 Legnaro, Italy

**Keywords:** Australia, *Acacia*, *Eucalyptus*, *Corymbia*, processionary moth, transplant

## Abstract

**Simple Summary:**

Host specificity in an Australian species of processionary moth was studied over three years in the eastern Australia states of Queensland and New South Wales. The processionary moth *Ochrogaster lunifer* is known as a species complex, characterised by several nesting forms coexisting in the same geographical area. The canopy nesting forms associated with acacias (*Acacia* spp.) and eucalypts (*Eucalyptus* spp. and *Corymbia* spp.) were recently shown to be genetically distinct, and they were here tested for host specificity by reciprocal transplant experiments. Egg masses and subsequent offspring were moved within and among putative hosts in field settings. Early larval colonies performed significantly better on their natal hosts than on the transplanted ones, with no difference observed for mature larval colonies. The genetic separation identified in previous work is, thus, confirmed by these ecological observations on host specificity, stressing the importance of host specialization in the ongoing speciation process.

**Abstract:**

The ‘mother knows best’ hypothesis is tested in a species of processionary moth feeding on acacias and eucalypts in Australia. The processionary moth *Ochrogaster lunifer* (Lepidoptera: Notodontidae; Thaumetopoeinae) is a social caterpillar living in large colonies on a number of tree and shrub species. Five nesting types—canopy, trunk, tree-hugger, hanging, and ground—have been described, and this study deals with canopy nesters on various species of acacias (*Acacia* spp.) and eucalypts (*Eucalyptus* spp. and *Corymbia* spp.). Reciprocal transplant experiments conducted over three years confirm the ‘mother knows best’ hypothesis, as colonies performed better on the natal host plant than on the recipient ones. Young first instar larvae were less likely to establish on a non-natal host than the mature larvae, and all acacia-sourced canopy egg masses failed to establish on eucalypts. Large larvae were able to establish on transplant hosts. This suggests a strong preference–performance link at what is likely a species level, confirming preliminary results recently published on genetic divergence. Canopy nesting forms also have a lower realised fecundity than the ground nesting form on acacias from the same geographic area, but higher than another canopy nesting form from western Australia. Further observations on ecological and genetic traits are required to draw conclusions about the separation of lineages in the canopy nesting form of *O. lunifer*, by including populations from other parts of the range for both the herbivore and the host plants.

## 1. Introduction

There has been a long-standing debate on host specificity in Lepidoptera. The ‘mother knows best’ hypothesis posits that females select hosts which maximise fitness [1]. Various reviews have garnered mixed results for and against [2,3,4], with the outcome depending on native versus exotic plants and numerous other factors (age of plant, damage, etc.) [4]. Simply observing eggs on a plant and following the survival of the offspring is not a sufficient test of the hypothesis. Field experiments using common garden plants enable host selection and subsequent survival to be measured at a number of sites at one time. Reciprocal transplant experiments offer another direct test, and we used this approach here to investigate host specificity in a widespread and locally common species of processionary moths.

The processionary moth, *Ochrogaster lunifer* Herrich-Schäffer, 1855 (Lepidoptera: Notodontidae; Thaumetopoeinae), is a species whose caterpillars live socially in large colonies on a number of tree and shrub species across Australia [5]. Eggs are laid in egg masses that normally include all the progeny of one female moth [6,7], and subsequent larvae are highly gregarious, feeding as a group through all immature stages. Host plants can be completely defoliated during outbreaks, and late instar caterpillars produce urticating setae that are a threat to animal and human health [8,9,10]. 

Common [5] suggested that *O. lunifer* is a species complex, based on different morphology and behaviour of the immature stages, and in particular the nesting type. Perkins et al. [11,12] provided the first comparative study on the nest types and how they relate to the veterinary importance of the species. There are five nesting types, namely canopy, trunk, tree-hugger, hanging, and ground nesters. Previous observations by van Schagen [8,9] were related to a canopy nesting form in south-west West Australia, while Floater [6,13,14,15,16], and Floater and Zalucki [17,18] provided an extensive review of the biology and behaviour of ground-nesting populations in coastal south-eastern Queensland. Perkins et al. [19] characterised the life history of a trunk (tree-hugger) nesting form restricted to *Corymbia tessellaris* (Myrtaceae). The species genetic status of *Ochrogaster* has recently been examined by Mather et al. [20], who suggested at least three genetic groups or species, associated with different nesting habits and host plants. 

Larval *O. lunifer* have a relatively narrow host range, feeding mainly on selected acacia and eucalypt species, but hosts in other families have been recorded. Acacias or wattles (*Acacia* spp., Fabaceae) and eucalypts (*Eucalyptus* spp. and *Corymbia* spp., Myrtaceae) have been found to be the most common hosts of *O. lunifer* [6,8,9]. According to Atlas of Living Australia (https://www.ala.org.au/ accessed on 29 March 2023), there are nearly 1000 species of *Acacia*, making them the largest genus of vascular plants in Australia [21,22]. Of these, 31 species have been identified as hosts. There are over 700 known species of *Eucalyptus* trees, shrubs, and mallees in Australia, and 13 have been identified as *O. lunifer* hosts. Furthermore, 4 of the approximately 113 species of *Corymbia* have been identified as *O. lunifer* hosts and, until the mid-1990s, they were classified as *Eucalyptus* species. Australia supports 92 million hectares of eucalypt forest and 9 million hectares of acacia forest, so it is reasonable to expect that there are many more *O. lunifer* host species yet to be documented. Other hosts include species of Sapindaceae and Caesalpiniaceae [7].

The objectives of this paper are to explore the host specificity of the canopy nesting forms occurring in Queensland and New South Wales on two major host types, acacias and eucalypts, by comparing the realised fecundity on those host plants and testing the preference–performance hypothesis by reciprocal transplant of egg masses and larval nests. 

## 2. Materials and Methods

### 2.1. Egg Mass and Larval Nest Collection

**Figure 1:**
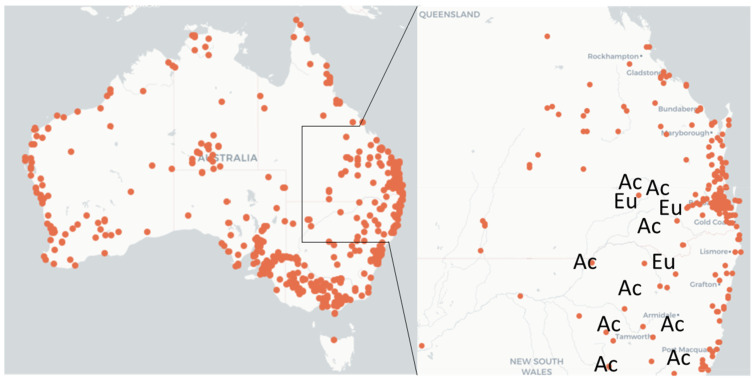
Map of Australia with the indication of the occurrence of *Ochrogaster lunifer* (orange circles) (from Atlas of Living Australia: https://www.ala.org.au/ accessed on 29 March 2023) and the location of the field sites (Ac = acacias, Eu = eucalypts) over an area of about 500 × 250 km. Field sites did not coincide with the finding sites reported in the map, although they could be close to them.

Sampling was conducted at 12 sites across Queensland and New South Wales (2015–2018, inclusive) and included at least 10 host plant species, from which 373 egg masses and 63 nests were collected (Figure 1, Table 1).

**Figure 2:**
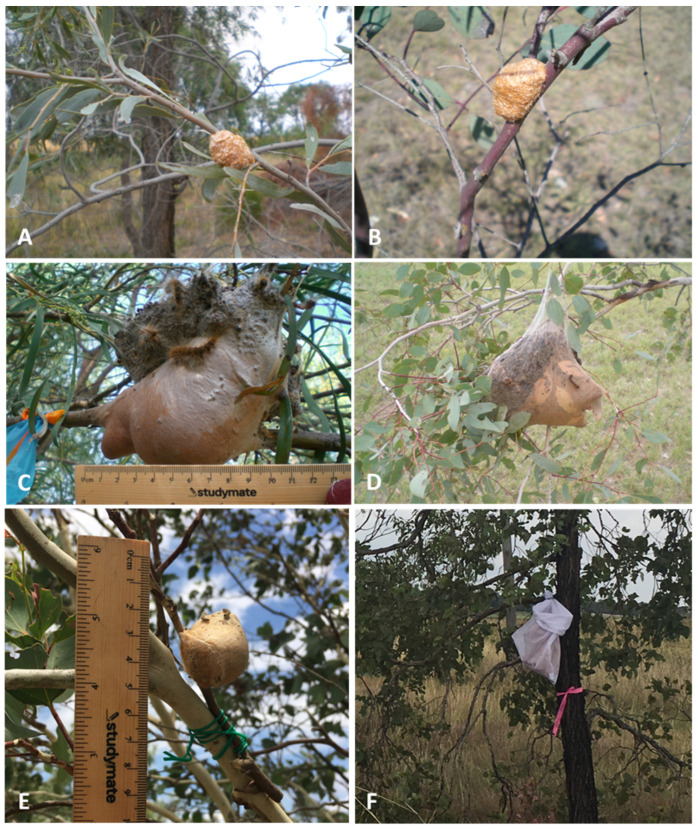
Egg masses and mature nests of the canopy nesting of *Ochrogaster lunifer*. (**A**). Egg mass on *Acacia pendula* near Moree, NSW 2015, (**B**). Egg mass on *Eucalyptus orgadophila* near Pittsworth, Qld 2015, (**C**). Mature nest on *Acacia stenophylla* near Malu, Qld 2016. (**D**). Mature nest on *Eucalyptus orgadophila* near Pittsworth, Qld 2016. (**E**). Transplant of an egg mass on *E. orgadophila*. (**F**). Transplant of a mature nest on *E. melanophloia*.

Between October and December in each year of the study, newly deposited egg masses were located randomly by inspecting the canopy of trees that contained nest remnants from previous generations of moths. The same amount of searching time was used in all years at each site (ca 1 hr). The egg masses stand out on the host because of their conspicuous size and lighter colour, whereas nests are less obvious to an observer (Figure 2A–F) and may often persist for several months after the larvae abandon them to pupate in the soil. The egg masses were collected by clipping by secateurs each side of the branch upon which they were laid. Some were frozen for later analysis of realised fecundity and others were retained in ventilated plastic containers for the experiments by re-attaching them to another host using plastic-coated garden wire. Between February and April in each year of the study, canopy nests with resident caterpillars were collected from a subset of locations (Table 1); the same amount of time searching was also invested for each site in each year. As both egg masses and nests contain urticating setae, protective gear was used during the process.

### 2.2. Realised Fecundity

Egg masses were randomly chosen from the frozen egg masses taken from the two host plant groups and used to estimate the total number of eggs. The moth scales covering the eggs were carefully removed while frozen and then immediately disposed of to avoid contact with the urticating scales and hairs. The eggs were bathed in ethanol to dissolve the glue holding them together, and were then counted individually.

### 2.3. Egg Mass Transfers

To test the plasticity of dietary preferences of the first larval feeding stage, recently deposited egg masses were transferred between host species and locations during the three development seasons. Unhatched egg masses were selected based on having similar sizes, rejecting small egg masses. The recipient trees were selected based on the absence of egg masses, presence of a suitable number of leaves for feeding, and accessibility. The choice in several cases was limited, and it was not possible to standardize the number of egg masses deployed among localities and tree species in the same group. The host combinations and the number of egg masses translocated are listed in Table 2.

Egg masses were clipped and attached to another tree branch at the same height using plastic-coated garden wire (Figure 2E). Controls were established by moving egg masses back to the collection host or to another tree of the same species if the original tree had inaccessible eggs masses higher in the canopy. All egg masses were labelled and tagged with fluorescent tape for ongoing monitoring of their fate. Due to different abundances of egg masses among sites in the three years of the study, the number of egg masses used in each year varied considerably. In 2015–2016, 50 canopy egg masses were collected from the Pittsworth, Foxes Lane, Edderton Road, and Gulargambone field sites for transfer to other hosts in the Hunter Valley (HV in Table 2), western NSW and south-east Qld. Many more masses were collected, frozen, and used for counting and measuring eggs. In 2016–2017, 61 egg masses were collected from the Pittsworth and Hunter Valley field sites to be transferred to alternative hosts in south-east Qld. In 2017–2018, 112 egg masses were collected from the south-east Qld and Hunter Valley field sites and transferred to alternative hosts in south-east Qld and western NSW (Table 2). The establishment of the colonies was assessed by periodic checks of hatching and nest formation. A colony was considered as established when a solid nest containing larvae of the third and fourth instar were detected. The absence of a nest denoted failure.

### 2.4. Larval Nest Transfers

To test the plasticity of dietary preferences of older caterpillars, established nests were transferred between host species and locations during the three development seasons. Nests were selected based on similar average size (>80 individuals), as well as being active and easy to access. The host combinations are listed in Table 2. Canopy nests were often difficult to remove from the original host without damage or with sufficient support webbing to allow attachment to another host. Nests and a short length of their branch were clipped and placed into large paper bags, then stapled closed to prevent caterpillar escape. Each nest was attached to the alternative host tree using plastic-coated garden wire (Figure 2F). The recipient trees were selected based on the absence of caterpillar nests and other defoliating agents, the presence of a suitable number of leaves for feeding, and accessibility. The choice in several cases was limited, and it was not possible to standardize the number of colonies deployed among localities and tree species in the same group. In 2015–2016, 21 nests were collected from *A. pendula* at Foxes Lane and Gulargambone and transferred to the canopies of *A. salicina* at Oakey and *Eucalyptus* sp. at Gulargambone. Seven nests were collected from *Eucalyptus* sp. at Allora and transferred to *A. stenophylla* at Malu. Controls in *A. pendula* were established at Gulargambone and Foxes Lane by clipping nests and attaching them to other nearby *A. pendula*. In 2016–2017, continuing heat waves during January and February caused many of the developing nests at the Qld and NSW field sites to be abandoned. The nests that survived contained a reduced number of resident caterpillars, resulting in fewer nest transfers. In 2017–2018, 20 nests were collected from defoliated *E. orgadophila* trees around Allora and transferred to the same or other host plants at other sites in south-east Qld. In addition, during a mass migration of late instar larvae from defoliated eucalypt (*E. orgadophila*) trees near Allora in early 2018, several thousand caterpillars were placed into large paper bags, about 200 larvae each, which were then stapled closed to approximate a nest and left to settle overnight. The paper bags were placed into pillowcases to provide support, and then attached to alternative host trees with holes made to allow caterpillar movement (Figure 2F). The establishment of the colonies was assessed by periodic checks of expansion of the nest, or formation of a new nest in the case of the bagged caterpillars. A colony was considered as established when webbing extended away from the nest and feeding activity was detected.

### 2.5. Data Analysis

Realised fecundity was compared between the two host plant groups (acacias and eucalypts) and years by the analysis of variance followed by pairwise comparison (Tukey test) after testing for normal distribution by χ^2^ test. The number of established and non-established colonies after both egg mass and larval nest transfers were tested against the null hypotheses of no difference between the paired samples by χ^2^ test, granting full independence to each tree within a group of host plants, irrespective of the species to which it belonged. All tests were carried out with Microsoft^®^ Excel^®^ for Microsoft 365, and with R (a language and environment for statistical computing, https://www.R-project.org/ accessed on 23 April 2023).

## 3. Results

### 3.1. Realised Fecundity

**Figure 3:**
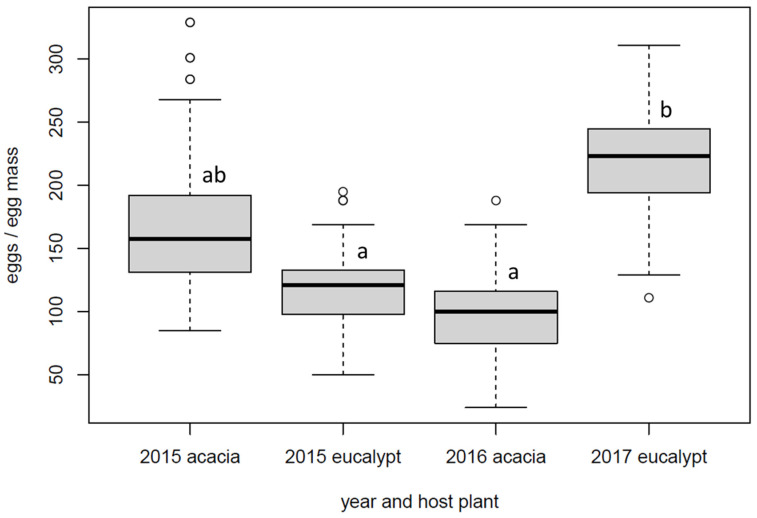
Boxplot of realised fecundity of *Ochrogaster lunifer* female moths in different years and host plant groups. Thick horizontal bars indicate the median, grey boxes represent the interquartile range, vertical bars represent the min–max values, and circles represent the outlier values. Different letters imply significant differences in pairwise comparisons (Tukey test, *p* < 0.05).

The egg masses used for the assessment of realised fecundity across the three years were 94 from acacias and 56 from eucalypts, but their abundance in the field varied greatly between years. Of the total number of egg masses, 101 were collected in 2015 on both hosts, 17 in 2016 only on acacias, and 32 in 2017 almost exclusively on eucalypts. Overall, the realised fecundity was normally distributed (χ^2^ test, *p* > 0.05), and the analysis of variance indicated significant differences in the dataset. While acacias and eucalypts did not differ significantly in realised fecundity (acacias: 156, SD 59.5; eucalypts 175, SD 64.6) (Tukey test, *p* > 0.05), there was a significant difference among the years for eucalypts but not for acacias (Tukey test, *p* < 0.01 and *p* > 0.05, respectively) (Figure 3).

### 3.2. Egg Mass and Mature Nest Transfers

**Figure 4:**
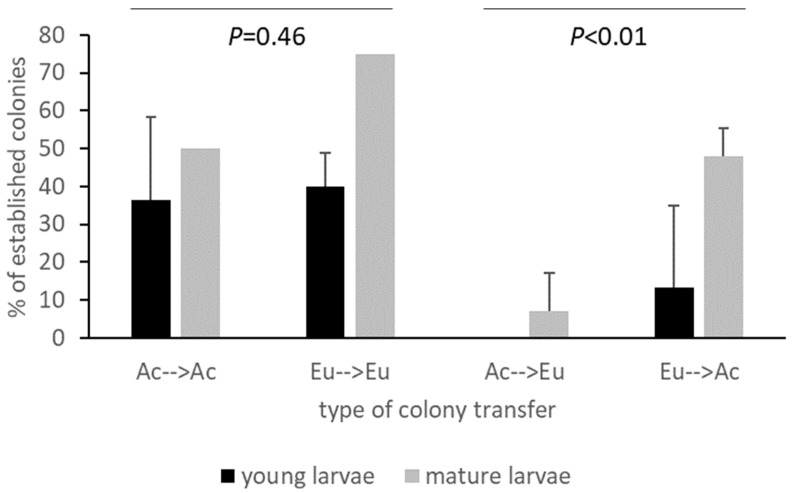
Establishment of colonies of *Ochrogaster lunifer* after transplant from the natal host plant groups (Ac = acacias, Eu = eucalypts) to recipient trees of the natal and non-natal host plant groups, for early instar and late instar larvae. Vertical bars indicate standard deviation. No standard deviation is available for late instar larvae, as nests were available only in one year. The lack of data for young larvae from acacias to eucalypts is due to total mortality of the early instar larvae.

The transplant experiments were largely dependent on the availability of egg masses and nests in different years. Nest availability was more affected than egg masses by the heat waves of 2016–2017, so no nest transplant experiments were conducted in that year. The overall survival of the transplanted colonies was 20% when starting with the egg masses and 52% with the nests. When averaging the results of the transplant experiments among the years of the study, colonies performed equally well on their original and transplanted natal hosts (χ^2^ test, *p* = 0.46) (Figure 4). However, in the crossed transplants between the two host groups, they fared better on their natal hosts than on the new ones (χ^2^ test, *p* < 0.01) (Figure 4). The difference was mainly explained by younger larvae hatched from the egg masses, while it was less significant for the mature larvae from the nest transplants. Colonies having acacias as natal host plants were significantly less likely to accept eucalypts (χ^2^ test, *p* < 0.01), especially as young larvae, for which not a single colony established on eucalypts. This was similar for mature larvae, although there was large variation among years (Figure 4).

## 4. Discussion

The results of the reciprocal transplant experiment confirm the ‘mother knows best’ hypothesis [1], although this is surprising as we are theoretically dealing with the same species on native host plants. The doubts cast by Common [5] about *O. lunifer* being a single species seem to be supported by our results, indicating that the canopy nesting form of *O. lunifer* on acacias could be a taxon different from that associated with eucalypts. Whether they are just diverging populations or reproductively isolated species should be confirmed by further ecological and genetic analyses. The preliminary results of Mather et al. [20] on the occurrence of at least three genetic groups or species in *O. lunifer* are supported by the ecological data presented here, and open the way to rediscuss the whole group at a larger geographic scale and including more of the many hosts described so far for the species.

Differences between the canopy nesting forms on acacias and eucalypts can be seen at different levels and, as such, compared with what is known for other nesting forms. Canopy nesting forms have a lower realised fecundity than the ground nesting form on acacias from the same geographic area where Floater [6] recorded egg mass size of 150–550 eggs (mean ca 268, SD 95, n = 24) and Uemura et al. [23] recorded 67–479 eggs (mean ca 301, SD 97, n = 65). Perhaps the difference reflects the greater distance this form must travel to garner its first meal, as the egg masses are laid at the base of the tree. The first instar of the ground nesting form of *O. lunifer* does not feed. It is the second instar that ascends the host tree to establish a feeding site and returns to the ground to build and maintain a nest, incurring a high mortality [17]. A comparison of the egg size between the ground and canopy nesting forms, as well as a thorough analysis of the behaviour of first instar in canopy nesting forms, would shed light on this aspect of the life history. Interestingly, a form of canopy nesting *O. lunifer* studied by Van Schagen et al. [8] in West Australia had only 75 eggs per mass, although only 6 egg masses were considered. The lower fecundity of the canopy compared to the ground nesting form is related to the female body size, as already pointed out by Floater [6].

In the canopy nesting forms considered in this study there was a clear variation of egg mass abundance and realised fecundity across time, with a marginal difference between the two host plant groups. The year was more important, with higher values of realised fecundity in 2015 and 2017 than in 2016. However, in 2015, egg masses were more frequent on acacias, while they were more frequent on eucalypts in 2017, documenting how the dynamics could be specific to the host plant. Although our study is lacking standardization on species within host groups and years due to the limited availability of trees in suitable conditions for sampling, it appears that the abundance and fecundity dynamics could be governed by the availability of resources in terms of plant quality or by the activity of natural enemies [24]. These two factors have been shown to be important for the establishment of colonies in previous studies on *O. lunifer* [8,17]. In particular, the interaction with weather (droughts or heat waves, such as the one observed in 2017) seems to be very important [15], and it could perhaps explain why there were independent dynamics on the two host plant groups, assuming that acacias and eucalypts respond in a different way to climatic variables [25]. Observations over a longer time periods could clarify the apparently contrasting response of the forms associated with different host plant groups. 

Colony performance was generally lower for egg masses than for nests, indicating that the establishment of the newly hatched larvae is a critical phase, even when they are moved within the same host plant. Although we note that the level of establishment is comparable to the ground nesting form determined by life table studies. Floater and Zalucki [17] found that of 492 egg cohorts monitored, 82% went extinct, mainly in the egg and early instar stage. Our transplants resulted in a better establishment (extinction rate of 65%), particularly when moved to the same host group, and again suggest that the shorter distance that larvae have to travel may influence their survival. In general, colonies performed better on the natal host plant than on the recipient ones, and the young larvae were less likely to establish on a non-natal host than the mature larvae. All acacia-sourced canopy egg masses failed to establish on eucalypts. This does suggest a strong preference–performance link at what is likely a species level. The apparent asymmetric performance of established larvae, particularly the better establishment of eucalypt to acacia versus acacia to eucalypt suggests that feeding on Myrtaceae requires specialised detoxification of the contained secondary compounds (terpenoids, phenolics), as shown by Schmidt et al. [26] for sawflies. That larger larvae could establish on novel hosts is perhaps not surprising for a defoliating insect—the larger children do not care, as has been observed in the related oak processionary moth *Thaumetopoea processionea* [27]. During outbreaks, larvae defoliate their hosts, and large larvae will move considerable distances in characteristic long processions to locate a new host [14]. Being able to accept other hosts would be advantageous, particularly if favoured hosts have been defoliated during outbreaks. Large larvae and nests of the acacia ground nesting form are often found completing development on *Casuarina* spp., although egg masses are rarely observed [6], (unpublished observations). Whether *O. lunifer* follows the Hopkins’ host selection principle [28] should be tested with specific experiments.

Here we have shown that the canopy nesting forms of *O. lunifer* on acacias and eucalypts are somewhat diverging in relation to the host plant groups. Specialization on host plants is perhaps a precursor for local adaptation and a possible driver of speciation. However, we did not assess growth performance and final survival on the different hosts and did not control for possible predation effects on the two groups of host plants. Such experiments should be carried out to further test the separation of lineages in the canopy nesting form of *O. lunifer*, also including populations from other parts of the range for both the herbivore and the host plants.

## Figures and Tables

**Table 1:** Location of field sites in Queensland (Qld) and New South Wales (NSW) where egg masses and nests of *Ochrogaster lunifer* were collected. Sites are listed alphabetically (HV = Hunter Valley).

Location	Co-Ordinates	Host Species	Egg Masses (n)	Larval Nests (n)
Allora, Qld	27°59′00″ S 151°57′33″ E	*E. orgadophila*	92	13
Ashford, NSW	29°10′12″ S 151°09′49″ E	*E. melanophloia*	8	0
Bylong Valley Way HV, NSW	32°20′39″ S 150°34′40″ E	*A. pendula*	20	0
Edderton Rd HV, NSW	32°19′40″ S 150°49′19″ E	*A. hakeoides*	28	0
Foxes Lane Moree, NSW	29°00′15″ S 150°01′26″ E	*A. pendula*	80	10
Gulargambone, NSW	31°18′50″ S 148°16′05″ E	*A. pendula*	10	0
Jerrys Plains HV, NSW	32°25′18″ S 150°41′48″ E	*A. pendula*	25	11
Jondaryan, Qld	27°22′19″ S 151°35′25″ E	*A. pendula*, *A. stenophylla*	5	0
Malu, Qld	27°20′22″ S 151°32′45″ E	*A. stenophylla*	0	5
Oakey, Qld	27°26′02″ S 151°43′16″ E	*A. salicina*	0	3
Pittsworth, Qld	27°42′30″ S 151°38′46″ E	*E. orgadophila*	105	18
Scone HV, NSW	32°06′53″ S 150°58′47″ E	*A. pendula*	0	3

**Table 2:** Egg mass and larval nest transfer within and between host species.

Natal Species	Recipient Species	Egg Masses	Larval Nests
*Acacia* (*pendula*, *salicina*, sp.)	*Acacia (concurrens*, *fimbriata*, *iteaphylla*, *pendula*, *salicina*, *stenophylla*)	56	10
*Acacia* (*pendula*, *salicina*, sp.)	*Eucalyptus (orgadophila*, sp.)	17	7
*Eucalyptus* (*orgadophila*, sp.)	*Eucalyptus (orgadophila*, *melanophloia*)	29	24
*Eucalyptus* (*orgadophila*, sp.)	*Acacia (concurrens*, *fimbriata*, *pendula*, *salicina*, *stenophylla)*	121	22

## Data Availability

The data presented in this study are available on request from the corresponding author. The data are not publicly available due to privacy.
